# The phenylalanine ammonia lyase (PAL) gene family shows a gymnosperm-specific lineage

**DOI:** 10.1186/1471-2164-13-S3-S1

**Published:** 2012-06-11

**Authors:** Ujwal R Bagal, James H Leebens-Mack, W Walter Lorenz, Jeffrey FD Dean

**Affiliations:** 1 Institute of Bioinformatics, The University of Georgia, Davison Life Sciences Bldg, Athens, GA 30602-7229, USA; 2 Department of Plant Biology, Miller Plant Sciences, The University of Georgia, Athens, GA 30602-7271, USA; 3 Warnell School of Forestry and Natural Resources, The University of Georgia, Athens, GA 30602-2152, USA; 4 Department of Biochemistry and Molecular Biology, The University of Georgia, Davison Life Sciences Bldg, Athens, GA 30602-7229, USA

## Abstract

**Background:**

Phenylalanine ammonia lyase (PAL) is a key enzyme of the phenylpropanoid pathway that catalyzes the deamination of phenylalanine to trans-cinnamic acid, a precursor for the lignin and flavonoid biosynthetic pathways. To date, PAL genes have been less extensively studied in gymnosperms than in angiosperms. Our interest in PAL genes stems from their potential role in the defense responses of *Pinus taeda*, especially with respect to lignification and production of low molecular weight phenolic compounds under various biotic and abiotic stimuli. In contrast to all angiosperms for which reference genome sequences are available, *P. taeda *has previously been characterized as having only a single PAL gene. Our objective was to re-evaluate this finding, assess the evolutionary history of PAL genes across major angiosperm and gymnosperm lineages, and characterize PAL gene expression patterns in *Pinus taeda*.

**Methods:**

We compiled a large set of PAL genes from the largest transcript dataset available for *P. taeda *and other conifers. The transcript assemblies for *P. taeda *were validated through sequencing of PCR products amplified using gene-specific primers based on the putative PAL gene assemblies. Verified PAL gene sequences were aligned and a gene tree was estimated. The resulting gene tree was reconciled with a known species tree and the time points for gene duplication events were inferred relative to the divergence of major plant lineages.

**Results:**

In contrast to angiosperms, gymnosperms have retained a diverse set of PAL genes distributed among three major clades that arose from gene duplication events predating the divergence of these two seed plant lineages. Whereas multiple PAL genes have been identified in sequenced angiosperm genomes, all characterized angiosperm PAL genes form a single clade in the gene PAL tree, suggesting they are derived from a single gene in an ancestral angiosperm genome. The five distinct PAL genes detected and verified in *P. taeda *were derived from a combination of duplication events predating and postdating the divergence of angiosperms and gymnosperms.

**Conclusions:**

Gymnosperms have a more phylogenetically diverse set of PAL genes than angiosperms. This inference has contrasting implications for the evolution of PAL gene function in gymnosperms and angiosperms.

## Background

Conifers have experienced large environmental and distributional changes during their evolution, dating back to the Mesozoic era [1]. To adapt to their diverse ecological habitats as well as the biotic and abiotic stresses associated with specific habitats, they have developed diverse and multi-layered chemical defense systems as a major component of their survival strategy [2]. Conifer defense systems synthesize a wide range of secondary metabolites upon pathogen attack. Central to these chemical systems, a wide variety of phenolic compounds, both low molecular weight toxins and highly polymerized physical barriers, such as in lignin, serve to prevent invasion by pathogens [3]. The precursors for many of these phenolic defense compounds are synthesized via the phenylpropanoid pathway [4].

The phenylpropanoid pathway has been extensively studied with respect to production of natural products, such as flavonoids, isoflavonoids, hydroxycinnamic acids, lignin, coumarins, stilbenes and a wide variety of other phenolic compounds. These products serve diverse functions in plants, including protection against biotic and abiotic stresses, cellular signalling, and UV protection, as well as mechanical support and response to low levels of iron and phosphate [5].

Phenylalanine ammonia lyase (PAL; E.C 4.3.1.5), the key enzyme linking primary metabolism of aromatic amino acids with secondary metabolic products in plants, has been extensively studied since its discovery by Koukal and Conn [6]. PAL plays a key regulatory role in controlling biosynthesis of all phenylpropanoid products. As the entry point into the pathway, PAL catalyses the non-oxidative deamination of phenylalanine to trans-cinnamic acid and ammonia. Trans-cinnamic acid, in turn, is the common precursor for the lignin and flavonoids biosynthetic pathways, which are highly complex and branched pathways [7]. Increased activity of PAL has been correlated with increased production of phenylpropanoid products [8], and levels of PAL activity vary with developmental stage, cell and tissue differentiation, and exposure to different stress stimuli [9-11]. PAL has been reported to be stimulated by infection, mechanical wounding, UV irradiation, drought stress and drastic temperature changes [12-14].

Until now, the gene content of conifer genomes has received less attention than angiosperm genomes despite the economic importance and ecological dominant of conifers in many terrestrial ecosystems [15]. Conifer genomes, at ca. 20 Gb on average, are larger than most angiosperm genomes. Yet in recent years, attempts to probe the genomic diversity of conifers have seen the development of such genomic resources as expressed sequence tag (EST) databases, cDNA microarray chips, and bacterial artificial chromosome (BAC) libraries, covering a handful of conifer species, notably loblolly pine (*Pinus taeda*) and white spruce (*Picea glauca*). Surprisingly, despite their large size, the structure of conifer genomes seems to be remarkably well conserved across well-diverged lineages. Chromosome number (12 or 13) is nearly the same in all conifer species (only three naturally occurring species of polyploidy conifer have been reported), and genetic mapping techniques have demonstrated substantial synteny across conifer species [16]. Although the organization of large conifer genomes has not yet been deeply studied, some gene families have been reported as being substantially larger in conifers than in angiosperms for which reference genomes are available [17], suggesting that gene duplication may be an important mechanism for genome expansion in conifers. Large multigene families have been suggested to be correlated with conifer genome size [18].

In contrast to numerous reports of PAL gene families in angiosperms, as well as a few other gymnosperms, only a single gene copy was reported to exist in the *P. taeda *genome [19]. An initial objective of this study was to assess whether uncharacterized PAL genes existed in the genomes of *P. taeda *and other conifers. Moreover, we were interested in assessing the duplication history of PAL genes in angiosperms and gymnosperms. Specifically, we wanted to characterize the timing of PAL gene duplication events relative to the origin of the conifers and the divergence of gymnosperms and angiosperms. The timing of these duplication events has implications for hypotheses concerning functional evolution within the PAL gene family.

Our results indicate that *P. taeda *possesses at least five (5) distinct PAL genes, and expression was demonstrated for at least four of these inferred genes. Phylogenomic analysis identified a diverse set of gymnosperm-specific PAL genes, with at least three conifer lineage-specific duplication events and two ancient duplications events predating the divergence of gymnosperms and angiosperms. These ancient duplications suggest a very different evolutionary history for the gymnosperm PAL gene family from that experienced by the family in angiosperms.

## Results

### PAL genes in Pinus taeda

For *P. taeda*, five distinct PAL consensus sequences were identified in *de novo *transcriptome assemblies performed using three different assemblers (Table 1). Complete coding sequences of ca. 725 amino acid residues were inferred for the pseudotranscripts of all five PtPAL genes. The number of ESTs identified for each of the five PAL genes varied nearly 30-fold between genes and between tissue-specific libraries, suggesting very different levels and patterns of expression for the different gene family members (*data not shown*).

**Table 1:** PtPAL (1-5) de novo transcriptome assemblies of P.taeda

**MIRA**^**1A**^	**Uniscript**^**2**^	**Uniscript length**^**3**^	**Total seq**^**4**^
PAL1/MIRA	P.taeda.JGI_rep_c1829	2081	295
PAL2/MIRA	P.taeda.JGI_rep_c1015	2660	392
PAL3/MIRA	P.taeda.JGI_rep_c9006	2826	142
PAL4/MIRA	P.taeda.JGI_rep_c4552	2474	155
PAL5/MIRA	P.taeda.JGI_rep_c10177	2269	62

**Newbler^1B^**	**Uniscript^2^**	**Uniscript** **length^3^**	**Total seq^4^**

PAL1/Newb	contig57512	3573	2924
PAL2/Newb	isotig35091	3022	606
PAL3/Newb	isotig22550	3110	506
PAL4/Newb	isotig41305	2538	279
PAL5/Newb	isotig35702	2278	87

**SeqMan NGen^1C^**	**Uniscript^2^**	**Uniscript length^3^**	**Total seq^4^**

PAL1/NGen	Contig347	3773	2746
PAL2/NGen	Contig13311	2889	560
PAL3/NGen	Contig5954	2370	223
PAL4/NGen	Contig26398	1798	154
PAL5/NGen	Contig50748	2277	75

^1A: ^miraEST (Mira) Version 3.0.5, ^1B^: Newbler Version 2.3, ^1C^: SeqMan NGen Version 3.0 (DNAStar), ^2: ^Contig name, ^3^: Contig lengths, and ^4^: numbers of sequences assembled to form a contig.

Because *de novo *assemblies generated in the absence of a reference genome sequence are susceptible to misassembly, we compared our contigs with sequences deposited in GenBank for conifer PAL genes that had been cloned and sequenced in previous studies. The lengths of the pseudotranscripts returned from each of the three assemblers were found to be reasonable in comparisons with related sequences in GenBank. For example, the previously cloned loblolly pine PAL1 gene [GenBank: U39792.1] is 2435 bp in length and showed 100% sequence identity to our PtPAL1 assembly. This inferred transcript length also matched well with full-length cDNA transcripts for the four Arabidopsis PAL genes [GenBank: NM_129260, NM_115186.3, NM_120505.3, NM_111869.3], which ranged from 2463 to 2584 bp in length.

**Figure 1:**
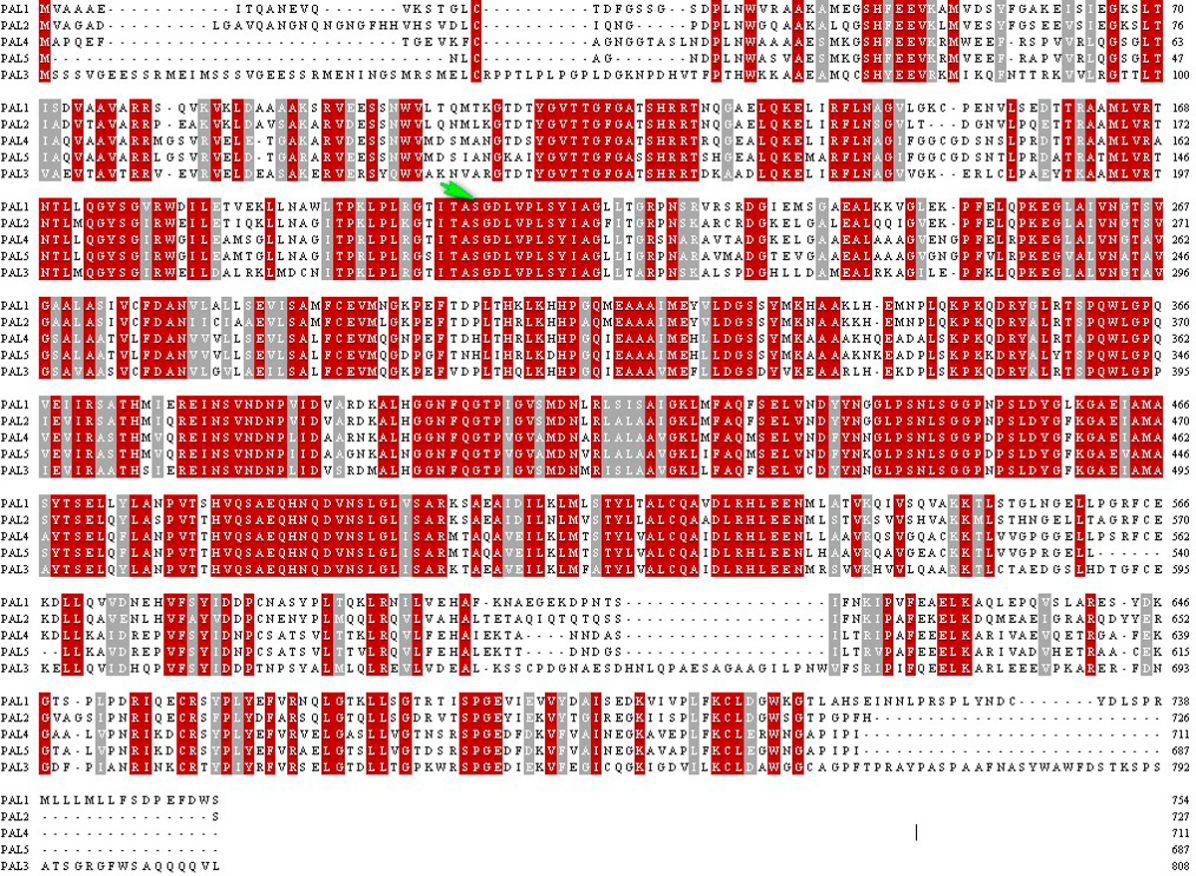
**Alignment between the five PtPAL genes in P. taeda**. Arrow indicates position of the conserved MIO region (Ala-Ser-Gly triad).

When compared to each other, PtPAL4 (Pteda28316) and PtPAL5 (Pteda34319) were quite similar at the amino acid level (93%), while PtPAL1 (Pteda1143311) and PtPAL2 (Pteda17307) exhibited just 86% similarity (Table 2). PtPAL3 (Pteda9006), the longest of the five sequences, showed the least identity to the other *P. taeda *PAL sequences (Figure 1).

**Table 2:** P.taeda PAL inferred amino acid sequence percent identity/similarity

**Gene id (**^**1**^**)**	PtPAL1	PtPAL2	PtPAL3	PtPAL4	PtPAL5
PtPAL1(754)	#	76/87	64/79	68/81	64/79
PtPAL2(727)		#	65/80	67/79	63/78
PtPAL3(808)			#	64/77	60/75
PtPAL4(711)				#	88/93
PtPAL5(687)					#

^1 ^Amino acid length.

The PtPAL1 sequence was found to be 98% identical to the genomic PAL gene sequence found on *P. taeda *BAC clone PT_7Ba2966L14 [GenBank AC241300.1]. Unlike angiosperm PAL genes, which include an intron, PtPAL1 and the PAL genes previously characterized in *P. banksiana *[20] lack introns.

### Validation of PAL cDNA sequence assemblies

Pine cDNA was amplified using gene-specific primer pairs corresponding to PtPAL1-PtPAL4. Amplification products of the expected sizes (300-450 bps) were detected as distinct bands on agarose gels (*data not shown*). These results confirmed expression of at least four members of the predicted PAL gene family in *P. taeda*. The sequence of the PtPAL5 proved too similar to PtPAL4 to allow for development of gene-specific primers that could discriminate between transcripts from the two genes. DNA sequencing of the amplified products confirmed the sequences inferred from our *in silico *assemblies.

### Sequence conservation

To detect sequence conservation between PAL genes from distantly related plant species, the inferred amino acid sequences of PAL genes from 25 species were aligned. In the alignment some of the PAL genes from gymnosperms showed higher homology to genes from non-gymnosperm taxa, which was also reflected in the subsequent phylogenetic analysis. Active sites residues, including those imparting substrate specificity, as well as those for catalysis and formation of the MIO [4- methylidine-imidazole-5-one] prosthetic group were clearly conserved (Figure 1), and as were additional residues previously noted as conserved in PAL proteins [21,22]. These observations strongly support the contention that all enzymes encoded by the genes included in these analyses bind and utilize the same substrate, phenylalanine.

### Phylogenetic analysis

**Figure 2:**
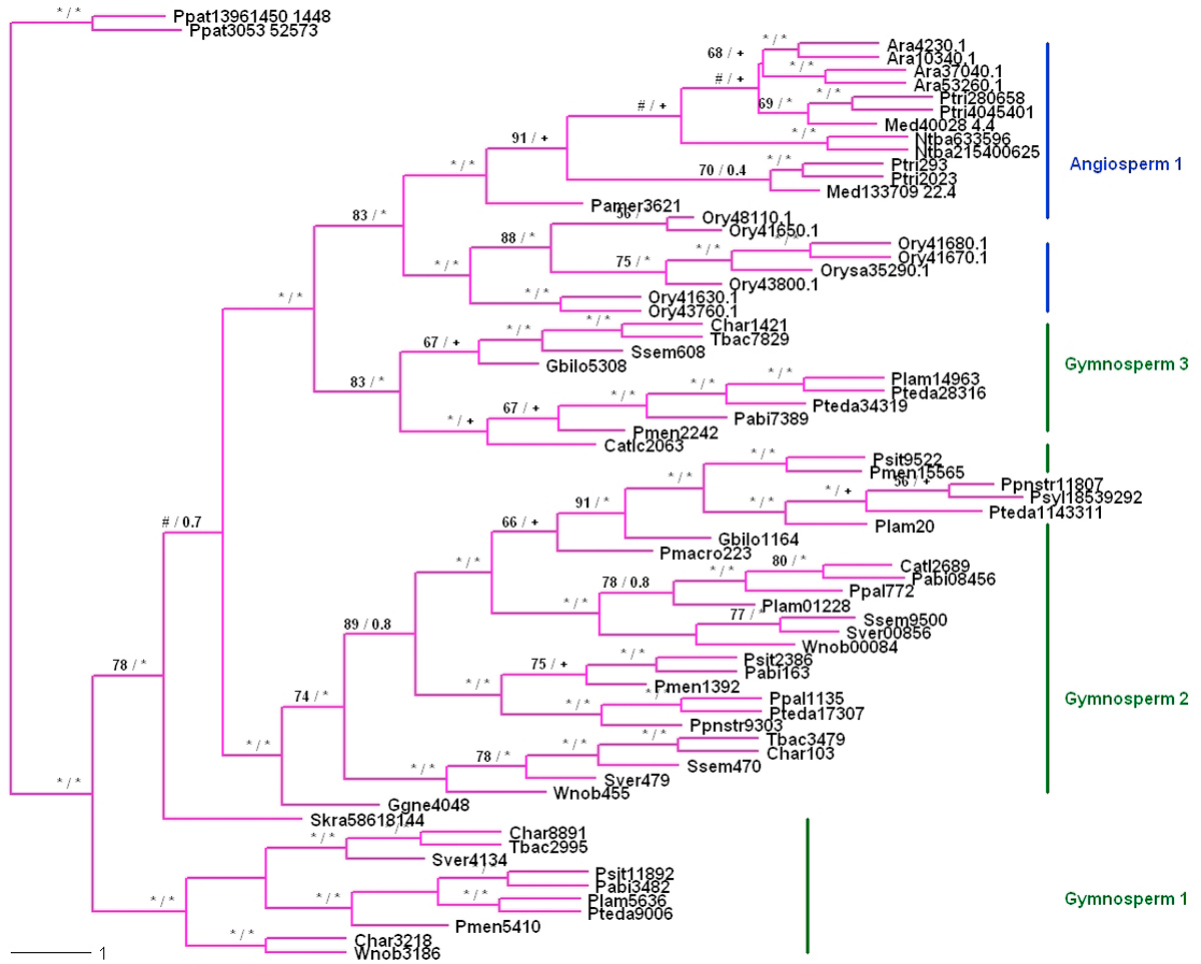
**Consensus tree of the Phenylalanine ammonia lyase gene family**. Numbers at nodes are nonparametric Bootstrap values (BS) from Maximum Likelihood (ML) and Posterior Probabilities (PP) from Bayesian Inference (BI), respectively, separated by a slash. Asterisks (*/*) symbol indicates [90-100]/[0.9 - 1.00] support values. The # symbol indicates BS values lesser than 50%. Plus (+) symbol indicate variation in branching patterns between the ML and BI consensus trees.

Phylogenetic analysis was performed to evaluate the evolutionary relationships among the 71 PAL sequences from 25 taxa selected for this analysis (Additional file 1). Trees were estimated from the multiple sequence alignment using Maximum Likelihood and Bayesian algorithms. In both analyses a PAL gene from *Physcomitrella patens *was used for the out-group (Figure 2). The consensus trees obtained using either method showed similar organization, with gymnosperm genes distributed among three distinct clades. One gymnosperm-specific clade was placed just above the out-group branches in the PAL gene tree. A clade with the remaining genes split into another gymnosperm-specific clade and a second clade containing both angiosperm and gymnosperm PAL genes. The high bootstrap values and posterior probability evidence provided strong support for the organisation of the gymnosperm genes into these three distinct clusters. Within the angiosperm PAL gene clade, monocot and eudicot gene clusters were each monophyletic as described in a previous report [23].

Because complete genome sequences are not yet available for pine and low gene expression levels often prevent sampling of particular mRNA sequences, the existence of additional PAL genes cannot be ruled out. It was clear from datasets for *Picea *cDNA sequences that additional PAL genes may exist in conifers since several homologous but incomplete *Picea *PAL gene sequences had to be removed from the collection prior to phylogenetic analysis because they were too short. PAL representation was similarly limited in the cDNA sets for other gymnosperms, but should improve as more sequences are added to the databases. Of particular interest for future studies will be functional analyses of gymnosperm PAL genes from all three gymnosperm-specific clades.

**Figure 3:**
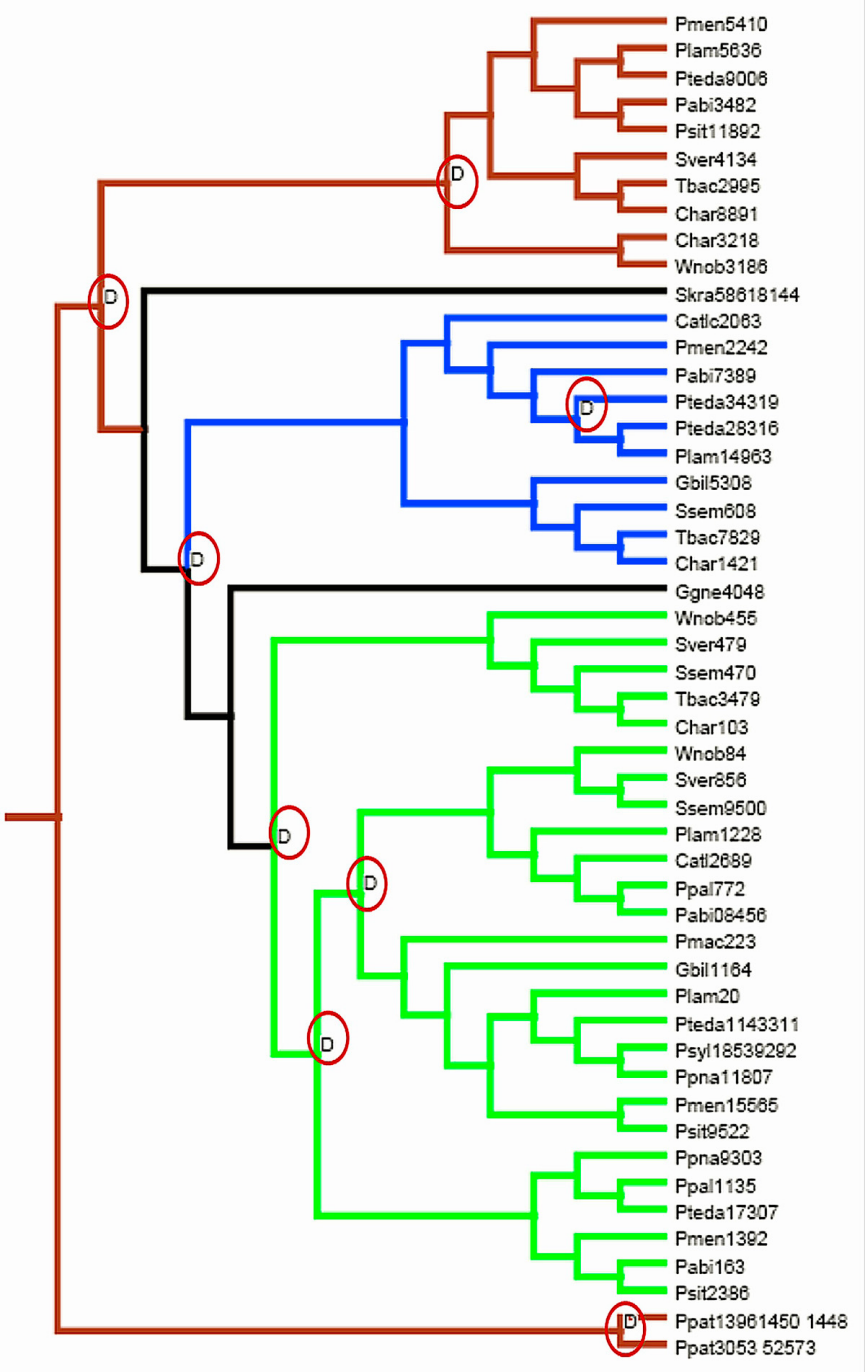
**NOTUNG: reconciled gene tree**. A reconciled gene tree with duplication events as obtained from Notung is depicted. Duplication nodes are marked with circles. The branch shading corresponds to the pattern of gymnosperms branching. The blue branch indicates gymnosperm sequences that clustered with angiosperm PAL genes. The green branch indicates a unique gymnosperm branch, while the brown branch indicates gymnosperm sequences clustering with sequences from basal taxa.

A species tree based on taxonomic information from the National Center for Biotechnology Information (NCBI) database was used to reconcile the gymnosperm section of the gene tree, keeping *P. patens *as the out-group (Figure 3). Notung version 2.6 [24] was used to infer the relative timing of speciation and duplication events. At least five duplication events were successfully traced in the ancestral lineages and confirmed on the basis of strong bootstrap support and posterior probability. Parsimony mapping suggests successive origins of three distinct gymnosperm PAL gene clades before the origin of the angiosperm clade. Ancestral seed plants had three distinct PAL genes which have been conserved in gymnosperms, but two of these ancestral genes were lost in the angiosperm lineage after divergence from the gymnosperms. In addition, PAL genes have also diversified more recently within the pines (Figure 2).

The oldest PAL gene duplication event evident in Figure 3 took place after the divergence of the vascular plants (Tracheophyta) and mosses, as represented by *Physcomitrella*. The second oldest duplication took place after divergence of the seed plants (Spermatophyta) and *Selaginella *(Lycopodiophyta). Following these duplication events, the duplicate copies of PAL were retained in the gymnosperms and all but one paralog was lost on the branch leading to the angiosperms. Further diversification of the PAL gene family from a single gene copy occurred within the angiosperms after the split of the dicots and monocots. The occurrences of independent lineage-specific duplications within the monocots and dicots have led to substantial elaboration of PAL gene families in various species of angiosperms.

At least three ancestral duplication events within the gymnosperms were suggested on the basis of high confidence values. Because of incomplete sampling and low branch support across the conifer species, duplication events close to the tips of the tree were not fully resolved. One duplication event was evident within the Pinaceae family, where one of the duplicate gene copies was found in closely related pine species (*P. lambertiana *and *P. palustris*), which had smaller EST datasets, but not in *P. taeda*.

## Discussion

Phenylalanine ammonia lyase, which belongs to the lyase class I super-family of enzymes [7], is a primary control point for the phenylpropanoid pathway, which in part explains the multigene families seen for PAL in almost all plants studied to date [10,25-27]. This study is the most extensive phylogenomic study so far for the PAL gene family, particularly with respect to conifers.

*De novo *transcriptome assemblies without a reference genome can lead to misassembly of contigs where transcripts are inaccurately joined together or single transcript can be split into two [28]. Three different programs were used to assemble the transcriptomes of *P. taeda *and 12 other conifers. We were able to identify five distinct PAL genes in all three *P. taeda *cDNA sequence assemblies. The contig lengths were comparable to those of cloned PAL genes available in the GenBank, suggesting no obvious errors in the assemblies.

The total number of sequences assembled to form each contig varied for all five PALs reflecting variation in their respective expression patterns [*data not shown*]. Differential expression patterns suggest that the various PtPAL gene products may be responsible for providing biosynthetic precursors to different phenylpropanoid branch pathways under different developmental conditions or in response to various external stimuli.

Apparently complete coding sequences were obtained for all five *P. taeda *PAL genes. Variability in the sequences was mostly associated with the terminal ends of the coding sequences. As PtPAL4 and PtPAL5 were 88% identical at the nucleotide level and clustered together on the same phylogenetic branch, they cannot be ruled out as allelic forms. Gymnosperm PAL genes were clustered into three clades. The origin of the most ancient clade is estimated to predate the origin of vascular plants (including *Selaginella*) while the other two clades originated by gene duplication within a seed-plant ancestor before the divergence of angiosperms and gymnosperms. This result suggests that PAL genes were lost on the branch leading to angiosperms.

The phylogeny of the PAL gene family identified in this study showed distinctive branching patterns for the monocot, dicot, and gymnosperm clades. The monocot-dicot split has been described previously [23]. In addition to ancient duplication events in a common ancestor of vascular plants and seed plants, respectively, distinct PAL genes clades within the monocots and eudicots point to lineage-specific diversification events within each of these taxa. The gymnosperm PAL clade that is sister to the angiosperm clade may include genes encoding for PAL isoforms that have similar functions or are regulated by similar developmental control mechanisms [29].

The existence of two additional gymnosperm PAL gene clades indicates maintenance of PAL genes in gymnosperms and loss of diversity in angiosperms [30]. The branching patterns within the conifer genes within these clades are in accordance with patterns reported previously for these species [1].

Duplication events have been an important theme in the evolution of the PAL gene family. At least five distinct duplication events can be identified in the PAL gene tree, with the oldest event following the divergence of *Physcomitrella*. Duplication events in the ancestral lineage, as well at the tip of the gymnosperm branch, suggest potential sources of functional variability [29]. Multigene families can be formed for a variety of reasons. It may be for production of additional trans-cinnamic acid for downstream metabolic pathways in these lineages; for instance, for increased expression of lignin biosynthesis in response to insect and pathogen attack [30]. Duplicate copies of these genes may encode different isoforms, or each duplicate copy may have a distinct expression pattern in terms of response to different physiological needs, such as tissue development or resistance to biotic and abiotic stresses [31]. Thus, in artichoke, three different PAL genes were suggested to play different roles in defense responses [32]. In Poplar, one PAL gene product was associated with formation of condensed tannins while another was associated with lignin production [23]. In tobacco, post-transcriptional regulation of one PAL gene in the family was reported, although the exact mechanism was not clear [33]. Early duplication events within a gene family, when compared to recent divergence events where genes from same species cluster together, have shown distinguishable biochemical, molecular and catalytic properties [26]. Based on this model, PtPAL4 and PtPAL5 may have resulted from a recent duplication event and may still serve overlapping functions (Figure 2). Likewise, as seen in other species, PtPAL genes that do not cluster together are more likely to encode PAL isozymes having unique functions, perhaps playing different metabolic role by producing different products under varying conditions.

## Conclusions

Five PtPAL genes were identified in cDNA assemblies for loblolly pine. The phylogenetic tree constructed using PAL gene sequences from 25 species including angiosperms, gymnosperm and basal taxa shows a very different evolutionary history for PAL genes in the gymnosperms, which may suggest different functional regulation. Reconciliation suggests early duplication events in the evolutionary history of PAL gene family as the root cause of phylogenetically separated genes rather than recent duplication events, which would lead to gene clustering.

## Methods

### PAL in conifer assemblies

A Community Sequencing Project undertaken at the US DOE Joint Genome Institute (http://www.jgi.doe.gov/) used 454 pyrosequencing to produce EST datasets for 12 conifer species, namely *Cedrus atlantica *(SRA023736), *Cephalotaxus harringtonia *(SRA023613), *Gnetum gnemon *(SRA023615), *Picea abies *(SRA023567), *Pinus lambertiana *(SRA023577), *Pinus palustris *(SRA023739), *Pinus taeda *(SRA023533), *Podocarpus macrophyllus *(SRA023741), *Pseudotsuga menziesii *(SRA023776), *Sciadopitys verticilliata *(SRA023758), *Sequoia sempervirens *(SRA023765), *Taxus baccata *(SRA023771), *and Wollemia nobilis *(SRA023774). All sequences are available from the Short-Read Archive (SRA) at GenBank.

Along with previously generated Sanger EST sequences available in GenBank, five cDNA libraries representing various tissues, treatments and genotypes of *P. taeda *yielded over 4 million reads used in these studies. Elongating shoot tissue cDNA libraries for the remaining conifer species were sequenced to yield from 0.4 to 1.2 million reads per species. The sequences were all assembled using three different assembly algorithms, namely Newbler Version 2.3 (454 Life Sciences), miraEST (Mira) Version 3.0.5 [34], and SeqMan NGen Version 3.0 (DNAStar). The consensus sequences along with their annotations from all the three assemblies, as well as such information as number of sequences aligned to form a contig and overall contig length, were retrieved from the Conifer DBMagic database [35].

Existing PAL sequences from *P. taeda *and other angiosperms available in GenBank were used as seeds to perform BLAST searches against the Conifer DBMagic database for novel PAL sequences from *P. taeda *and the other 11 conifers. Contigs with complete or near-complete coding sequence was selected for further analyses, while shorter sequences were discarded.

### Sequence verification

Since the assembled sequences were products of *de novo *assemblies, they were considered prone to error. To confirm that the sequences represented true gene products, experimental verification was performed by designing gene-specific primers for the PtPAL1-PtPAL4 consensus sequences and verifying the identity of amplified products by sequencing of the PCR amplimers.

The same assembled contigs used for the phylogenetic analysis were used as the basis for designing gene-specific oligonucleotide primers for PCR studies. A pair of PCR primers, Fwd ["AAGAACGCAGAAGGTGAGAAGG"] and Rev ["AGCATTTGAAGAGAGGGACTATGAC"], were designed to amplify 307 bp from PtPAL1 (Pteda1143311). In a similar fashion, Fwd ["CTGACTGAGACTGCCCAAATTC"] and Rev ["TCCTCCTGCCGTTTCCAATG"] primers amplified a 444 bp sequence from PtPAL2 (Pteda17307), Fwd ["TCAGAGTTGGGAACCGATTTG"] and Rev ["CTATTGATTCATTGTTGTTGGAACC"] primers amplified a 388 bp sequence from PtPAL3 (Pteda9006), and Fwd ["CCAATAACGACGCTTCTATCCTTAC"] and Rev ["CGCCGTTCCATCGCTCAAG"] primers amplified a 306 bp sequence from PtPAL4 (Pteda28316). The quality of these primers was assessed *a priori *using the program Beacon Designer 3 (PREMIER Biosoft International, Palo Alto, CA).

PCR amplification of PAL cDNAs synthesized from mRNA extracted from the stem tissues of *P. taeda *seedlings was performed in a 50 μl reaction volume. Reaction mixtures contained 1 μl of Taq polymerase, 2 μl of 10 mM dNTP, 4 μl of Optiprime 10× buffer, 3 μl of 5 mM primer and 10 μl of 1 ng/μl cDNA template was used for each gene-specific amplification reaction. Amplification was performed using a GeneAMP PCR system 9700 thermocycler (Applied Biosystems, Culver City, CA). The cycling conditions were 1 cycle of 95°C for 3 min followed by 40 cycles of 94°C for 30 secs, 55°C for 30 secs, 72°C for 90 sec, and 1 cycle of 72°C for 10 min. PCR products were purified using a DNA purification kit (Invitrogen Corporation, Carlsbad, CA) and dideoxy sequencing was performed using an Applied Biosystems 3730XL sequencer at the Georgia Genomics Facility (http://dna.uga.edu/).

### Taxonomic representation

Based on preliminary phylogenetic analyses, 25 representative taxa were selected for compilation of PAL genes, sequence alignment and tree reconstruction. The selected taxa (the number of PAL genes used from each species is shown parenthetically) comprised five dicotyledonous angiosperms, namely, *Arabidopsis thaliana (4), Medicago truncatula (2), Nicotiana tabacum (2), Persea americana (1) and Populus trichocarpa (4), and one monocot, Oryza sativa (8)*. Nineteen gymnosperm taxa were analyzed, including *Cupressus atlantica (2), Cephalotaxus harringtonia (4), Ginkgo biloba (2), Gnetum gnemon (1), Picea abies (4), Picea sitchensis (3), Pinus lambertiana (4), Pseudotsuga menziesii (4), Pinus palustris (2), Pinus pinaster (2), Pinus sylvestris (1), Pinus taeda (5), Podocarpus macrophyllus (1), Sciadopitys verticillata (3), Sequoia sempervirens (4), Taxus baccata (3), and Wollemia nobilis (3)*. Two non-seed plant taxa, the moss, *Physcomitrella patens (2)*, which also served as an out-group, and the lycopod, *Selaginella kraussiana (1)*, were also used for these analyses.

### Taxon sampling and phylogenetic analysis

The nucleotide sequences and corresponding amino acid sequences for the representative taxa were collected from various public databases, including GenBank, PlantGDB and PlantTribes [36-38]. The inferred transcript sequences for *C. atlantica, C. harringtonia, P. abies, P. lambertiani, P. macrophyllus, P. palustris, P. sylvestris, S. verticillata, S. sempervirens, T. baccata*, and *W. nobilis *were contigs assembled from cDNA datasets obtained by pyrosequencing. Using different angiosperm and gymnosperm PAL genes as seeds, outputs with expect-values (e-value) of 1e^-45 ^and below were selected for use in the study. The resulting dataset was further sorted and screened to remove possible contaminations resulting from assembly errors, sequences with length ≤ 50% of the complete CDS length, or putative allelic sequences sampled from the same species, i.e. those with nucleotide sequence identities ≥ 95%. Following the screening process, 71 sequences from 25 taxa remained for phylogenetic and molecular evolutionary analyses of the *PAL *gene family.

An initial multiple sequence alignment for the complete dataset was performed using MAFFT [39]. Multiple codon alignment corresponding to protein sequences was performed using PAL2NAL [40]. Molecular phylogeny estimates were derived using RAxML [41] and MrBayes [42] on a 2430 character sequence alignment. For the RAxML estimation, a generalized time-reversible (GTR) substitution model [43] with across-site rate variation modelled as a gamma distribution [44] and invariant sites (GTR+GAMMA+I), was used for nucleotide alignments. For amino acid alignment, the JTT [Jones, Taylor and Thornton] substitution model [45] with gamma distribution was used. Clade support was evaluated using 100 bootstrap replicates. For the MrBayes analysis, the GTR model was used with GAMMA correction and eight discrete rate categories. Analyses with MrBayes were performed over two runs, including four chains and three million generations per run. After 750,000 (25%) burn-in generations, trees were sampled every 300 generations and used to estimate posterior probabilities for each clade.

## List of abbreviations used

PAL: Phenylalanine ammonia lyase; PtPAL: PAL from *Pinus taeda*.

## Competing interests

The authors declare that they have no competing interests.

## Authors' contributions

JLM and JFDD conceived the general idea of the study; WWL carried out the assembly work. URB acquired the PAL sequences and performed the analysis on them. URB and JLM interpreted the data.

## Supplementary Material

Additional file 1 **Nucleotide sequences used for analysis**. The given file contains sequences downloaded from the public database for angiosperms and few of the gymnosperms. It also contains assembled consensus sequences for gymnosperms. These sequences were used for getting amino acid as well as codons for evolutionary analysis. Primers specific to PtPAL were designed using PtPAL1 to PtPAL4. Click here for file
